# Effect of WS_2_ Inorganic Nanotubes on Isothermal Crystallization Behavior and Kinetics of Poly(3-Hydroxybutyrate-*co*-3-hydroxyvalerate)

**DOI:** 10.3390/polym10020166

**Published:** 2018-02-09

**Authors:** Tyler Silverman, Mohammed Naffakh, Carlos Marco, Gary Ellis

**Affiliations:** 1 Escuela Técnica Superior de Ingenieros Industriales, Universidad Politécnica de Madrid (ETSII-UPM), José Gutiérrez Abascal 2, 28006 Madrid, Spain; tsilverman1@gmail.com; 2 Instituto de Ciencia y Tecnología de Polímeros (ICTP-CSIC), Juan de la Cierva 3, 28006 Madrid, Spain; cmarco@ictp.csic.es (C.M.); gary@ictp.csic.es (G.E.)

**Keywords:** inorganic nanotubes, PHBV, nanomaterials, morphology, crystallization kinetics

## Abstract

Nanocomposites of poly(3-hydroxybutyrate-*co*-3-hydroxyvalerate) (PHBV) and tungsten disulfide inorganic nanotubes (INT-WS_2_) were prepared by blending in solution, and the effects of INT-WS_2_ on the isothermal crystallization behavior and kinetics of PHBV were investigated for the first time. The isothermal crystallization process was studied in detail using various techniques, with emphasis on the role of INT-WS_2_ concentration. Differential scanning calorimetry (DSC) and polarized optical microscopy (POM) showed that, in the nucleation-controlled regime, crystallization rates of PHBV in the nanocomposites are influenced by the INT-WS_2_ loading. Our results demonstrated that low loadings of INT-WS_2_ (0.1–1.0 wt %) increased the crystallization rates of PHBV, reducing the fold surface free energy by up to 24%. This is ascribed to the high nucleation efficiency of INT-WS_2_ on the crystallization of PHBV. These observations facilitate a deeper understanding of the structure-property relationships in PHBV biopolymer nanocomposites and are useful for their practical applications.

## 1. Introduction

Over recent years, bio-based products have attracted increasing interest due to escalating environmental concerns and diminishing fossil resources [1]. Consequently, there is and has been a growing demand and interest, in both academic and industrial realms, to investigate biopolymers (i.e., polymeric material of non-fossil, biological origin) and develop strategies that can implement them for societal needs. Biopolymers can be broadly classified into two main categories: agropolymers, such as starch and other carbohydrates, proteins, etc., and biodegradable polymers, such as polyhydroxyalkanoates (PHAs), poly(lactic acid), etc. Biosynthetic polymers such as PHAs are linear, aliphatic polyesters that are produced by a microbial process in a sugar-based medium, where in certain bacteria they act as carbon (energy) storage banks [2]. A family of these materials from over 150 different monomers can be obtained with incredibly diverse properties [3]. Of these, polyhydroxybutyrate (PHB), polyhydroxyvalerate (PHV), and their copolymers poly(hydroxybutyrate-*co*-hydroxyvalerate) (PHBV), are commonly used matrices in bio and eco-composites [4]. Whilst PHB exhibits high stiffness and crystallinity, the incorporation of 3-hydroxyvalerate (HV) groups in a random copolymer with 3-hydroxybutyrate (HB) is a strategy used to increase the flexibility and processing capabilities of the polymer, reducing stiffness, melting point and crystallinity of the copolymer on increasing the HV content [3]. Despite improved thermal and mechanical properties, PHBV still presents some disadvantages, which include a narrow processing window, a slow crystallization rate, and low values of strain-at-break, along with a higher cost when compared with petroleum-based synthetic polymers [5]. To solve the aforementioned limitations, methods such as physical blending or chemical structure design combined with processing conditions can be applied to improve the performance of PHA products [3,6].

Nanocomposite strategies have been suggested to overcome the inherent shortcomings of biopolymer-based materials, and nano-biocomposites obtained by introducing nanofillers into biopolymers result in very promising materials, manifesting improved thermal and mechanical properties whilst maintaining material biodegradability, without introducing toxicity [6]. These find applications mainly in packaging, agriculture, and biomedical or hygiene devices, and represent an emerging alternative towards environmentally benign and economically viable chemical production [7]. Depending on the processing conditions from the melt into the solid state, biopolymeric materials may partially crystallize into a semicrystalline morphology that affects the aforementioned relevant properties. For this reason, numerous studies have been undertaken to characterize the crystallization behavior and to control the crystallinity, the crystallization kinetics, the spherulitic superstructure, or the crystal polymorphism, employing calorimetry and optical microscopy techniques [3,8]. In particular, calorimetry enables quantification of transition temperatures and enthalpies in isothermal and non-isothermal modes. Lorenzo et al. [8] have suggested a methodology for the minimization of possible errors associated with data manipulation in the measurement and analysis of conventional experimental DSC data. Isothermal crystallization experiments performed by DSC showed an increase in the crystallization kinetics of polycaprolactone (PCL) with increases in carbon nanotubes content as a consequence of the supernucleation effect [9]. Making use of fast scanning chip calorimeters and combining both approaches allowed them to shed further light on fundamental details of the polymer-crystallization process [10]. Furthermore, systematic studies of nucleation, crystallization, melting, and reorganization are made possible for a large number of polymers. In particular, promising research serving as a conceptual study to quantitatively approach the link between the condition of cooling the melt of crystallizable polymers, the formation of crystal nuclei and the cold-crystallization behavior have been successfully developed [11]. 

The enhancement of biodegradability, biocompatibility, thermal conductivity and mechanical proprieties of biopolymeric materials can be achieved by adding nanofillers [12,13,14,15]. On the other hand, the use of layered transition metal dichalcogenide nanofillers such as tungsten and molybdenum disulfide (WS_2_, MoS_2_) inorganic fullerenes (IFs) and inorganic nanotubes (INTs) [16,17] is expected to produce advanced nanocomposite materials [18,19]. As well as unique electronic and mechanochemical behavior, these novel nanomaterials show remarkable properties like high impact resistance and flexibility under tensile stress, excellent tribological behavior, superior fracture resistance to shockwaves, and simple and relatively inexpensive fabrication methods [20]. Recently, the incorporation of WS_2_ in polymer systems has demonstrated a range opportunities for many new applications. For example, IF-WS_2_ nanoparticles were used to produce advanced nylon-6 nanocomposites [21]. In particular, it was shown that introducing IF-WS_2_ nanoparticles into nylon-6 provoked a strong nucleation effect which induced changes in the crystal growth process. In the same way, the addition of low WS_2_ loadings strongly increased the crystallization rate of PHBV [22]. For these systems, drawing induced during melt crystallization process has been shown to vary the crystalline structure (i.e., from α to β) leading to improved mechanical properties in melt-spun bio-based PHBV fibers [23]. Similarly, WS_2_ nanotubes (INT-WS_2_) have been shown to improve the thermal, mechanical and tribological properties of biopolymers like poly(3-hydroxybutyrate) (PHB) [24] and poly(L-lactic acid) (PLLA) [25], and the bio-applied polymer poly(ether ether ketone) (PEEK) [26]. Additionally, from an environmental viewpoint, INT-WS_2_ have demonstrated much lower cytotoxicity than other nanoparticle fillers, such as silica or carbon black [27] and have shown promise with respect to biocompatibility in the case of salivary gland cells [28]. 

The present work continues progress in this field and is centered on the incorporation of INT-WS_2_ as nanoreinforcements to improve the processability and performance of PHBV. Scanning electron microscopy (SEM) observations revealed that an excellent dispersion of highly efficient INT-WS_2_ nucleating agents was achieved, leading to composites with substantially enhanced thermal and mechanical properties [29]. However, to date the influence of the nanofiller on the crystallization behavior and kinetics of PHBV under isothermal conditions has not been investigated. Here, this process is studied in detail using differential scanning calorimetry (DSC) and polarized optical microscopy (POM) techniques, with particular emphasis on the role of INT-WS_2_ concentration. The research reported provides a better understanding of the structure-property relationship of PHBV biopolymer nanocomposites, with an outlook towards extending their practical applications.

## 2. Experimental Section

### 2.1. Materials and Processing

The PHBV biopolymer employed, containing 2.0 wt % hydroxyvalerate (HV) with a reported Mw = 410 kg mol^−1^, was obtained in powder form from Goodfellow Cambridge, Ltd. (Huntingdon, UK) and used as received. The tungsten disulfide inorganic nanotubes (INT-WS_2_) were provided by NanoMaterials, Ltd. (Yavne, Israel) and used without chemical modification. Several formulations of PHBV/INT-WS_2_ (0.1, 0.5 and 1.0 wt %) nanocomposites were prepared [29]. The nanofiller was dispersed in a solution of PHBV in chloroform (HPLC grade, Sigma-Aldrich Química SL, Madrid, Spain), which was subsequently precipitated in methanol (HPLC grade, Sigma-Aldrich Química SL, Madrid, Spain), then filtered and dried in a vacuum oven at 50 °C for 24 h.

### 2.2. Characterization Techniques

#### 2.2.1. Differential Scanning Calorimetry (DSC)

The isothermal crystallization and melting behavior of the new nanocomposites were studied using a Perkin Elmer DSC7/Pyris differential scanning calorimeter (Perkin-Elmer España SL, Madrid, Spain). The instrument was calibrated for temperature and heat flow using high purity indium and zinc standard. A tau lag calibration of the instrument for different heating rates was performed using indium. The experimental and theoretical procedures used in this study are similar to those employed in our previous publications for PLLA/INT-WS_2_ [25] and nylon-6/IF-WS_2_ [21]. In this case, samples of 6–11 mg were placed in sealed 40 μL aluminum pans under a flowing nitrogen atmosphere. Before cooling, the samples were maintained for 5 min at 180 °C to erase any prior thermo-mechanical history and to assure maximum thermal stability of the components as well as the reproducibility of the results. Then the molten samples were cooled at fastest achievable rate of 64 °C min^−1^ to specific isothermal crystallization temperatures (*T_c_*) and maintained until crystallization was completed (i.e., complete return to baseline), and the heat evolved during crystallization was recorded as a function of time at selected *T_c_*. Pyris DSC7 kinetic software was used to obtain partial areas from the data points of the exotherm, corresponding to a given degree of the total crystalline transformation. The crystallinity of PHBV in the samples was determined, after normalizing for filler content, using a value of Δ*H_m_* for 100% crystalline PHBV (low HV content) of 146 J g^−1^ [3,30]. The isothermal crystallization step was followed by a heating step up to 180 °C at a rate of 5 °C min^−1^, and melting temperatures (*T_m_*) were taken as the peak maxima of the melting endotherms.

#### 2.2.2. Polarized Optical Microscopy (POM)

Polarized optical microscopy (POM) was used to investigate the spherulitic morphology of neat PHBV and the PHBV/INT-WS_2_ nanocomposites employing a Mettler FP-80HT (Mettler-Toledo SAE, Barcelona, Spain) hot stage on a Reichert Zetopan Pol polarizing microscope equipped with a Nikon FX35A 35 mm SLR camera. The isothermal crystallization cycles consisted in a 5-min hold period at 180 °C followed by rapid cooling at 20 °C min^−1^ to specific crystallization temperatures, *T_c_* = 110 °C, 112 °C and 122 °C. Samples were maintained at *T_c_* for enough time to allow the monitorization of the spherulitic growth process. 

## 3. Results

### 3.1. Isothermal Crystallization

**Figure 1:**
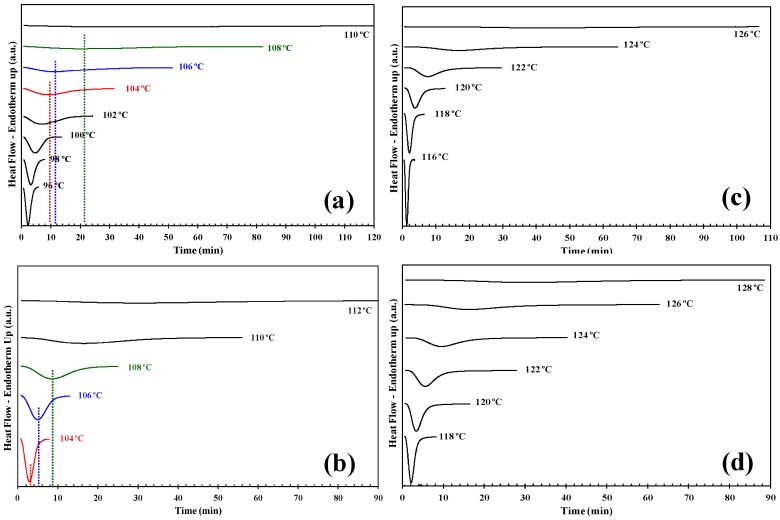
Differential scanning calorimetry (DSC) thermograms of isothermal crystallization of (**a**) PHBV; (**b**) poly(3-hydroxybutyrate-*co*-3-hydroxyvalerate)/ tungsten disulfide inorganic nanotubes (PHBV/INT-WS_2_) (0.1 wt %); (**c**) PHBV/INT-WS_2_ (0.5 wt %) and (**d**) PHBV/INT-WS_2_ (1.0 wt %) obtained at the indicated crystallization temperatures.

The physical and mechanical properties of semicrystalline polymers depend on the morphology, the crystalline structure and the degree of crystallinity. Much effort has been devoted to study the isothermal crystallization kinetics of new PHBV/INT-WS_2_ bionanocomposites, with a view to control the crystallization rate, degree of crystallinity and, consequently, its morphology and properties. In this respect, the isothermal melt crystallization kinetics of neat PHBV and its nanocomposites was investigated with DSC over a wide range of crystallization temperatures from 94 °C to 130 °C. The curves in Figure 1 indicate the total time for the complete crystallization process at the above-mentioned temperatures and are truncated at the time when no further crystallization was evident by DSC. Figure 1a shows that at higher *T_c_* more time is required to fully crystallize the pure PHBV sample. At lower *T_c_* the curves shifted to shorter times, indicating increased crystallization rates directly proportional to the isothermal crystallization temperature employed. The crystallization behavior of the nanocomposites with temperature was similar, Figure 1b–d. These results are consistent with the theory of crystallization kinetics, implying that as the supercooling (i.e., the difference between the melting and crystallization temperatures) increases, the crystallization rate accelerates and the crystallization exotherm becomes sharper, controlled in turn by the evolution of the number of crystal nuclei formed during crystallization process of the biopolymer matrix. In previous studies on the nucleation behavior of PLLA reported by Androsch et al. [11], it was shown that isothermal formation of crystal nuclei at high supercooling of the melt can be quantified by the analysis of crystallization at elevated temperature.

By comparing Figure 1a with Figure 1b–d, it is clear that at the *T_c_* the exothermic peaks for the nanocomposites are in all cases sharper than those for pure PHBV indicating that the INT-WS_2_ accelerates the crystallization process of the polymer in the nanocomposites. The reduction in the time to reach overall crystallization can be employed to describe this acceleration process. For example, the PHBV copolymer without INT-WS_2_ fully crystallized after approximately 120 min at *T_c_* = 110 °C, whereas for the 0.5 wt % nanocomposite material it took less than 4 min at *T_c_* = 116 °C (i.e., at a temperature of 4 °C higher than that of the pure polymer). From the data it can be seen that the incorporation of low INT-WS_2_ weight-fractions in PHBV nanocomposite allows the crystallization to take place at higher temperatures and over larger intervals (96–110 °C for PHBV, 116–126 °C for PHBV/INT-WS_2_ (0.1 wt %), 104–112 °C PHBV/INT-WS_2_ (0.5 wt %) and 118–128 °C for PHBV/INT-WS_2_ (1.0 wt %).

**Figure 2:**
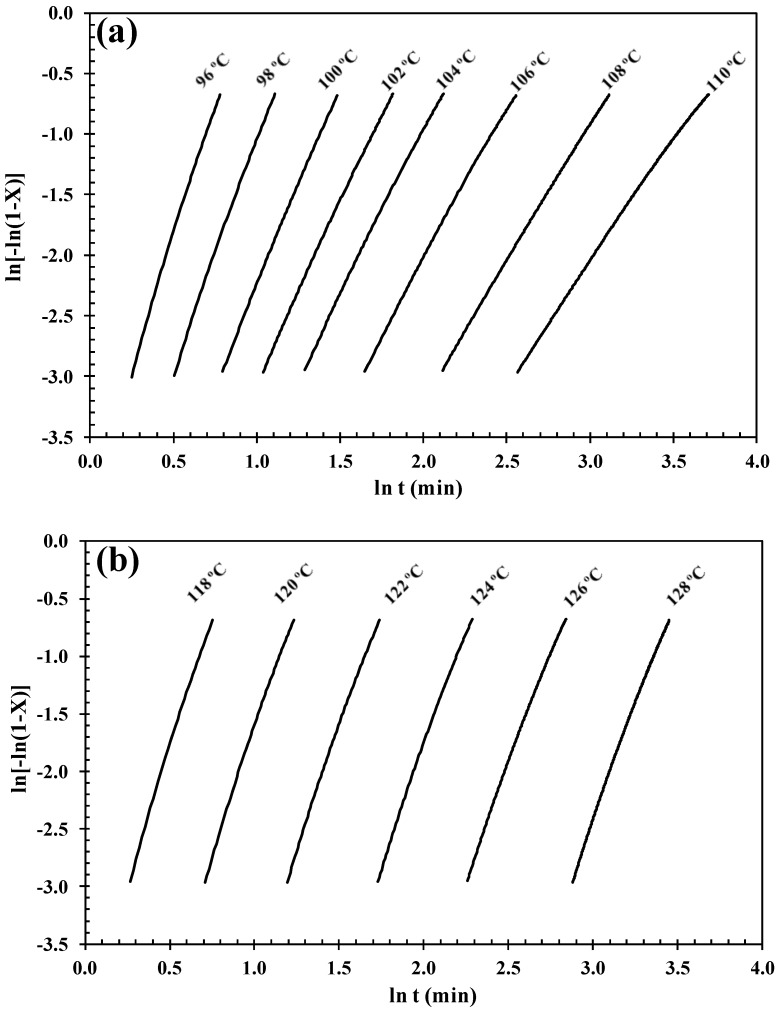
Avrami plots of the crystallization of (**a**) PHBV and (**b**) PHBV/INT-WS_2_ (1.0 wt %) as a function of the crystallization temperature (*T_c_*).

The relative crystallinity, *X*(*t*), can be defined by the following expression:

(1) $$X(t)=\frac{\int_{0}^{t}\frac{dH(t)}{dt}dt}{\int_{0}^{t\infty }\frac{dH(t)}{dt}dt}=\frac{\Delta H_{t}}{\Delta H_{\infty }}$$

where *dH*/*dt* is the heat flow rate; Δ*H_t_* is the heat generated at time *t*; Δ*H_∞_* is the total heat generated to the end of the crystallization process. The classical Avrami Equation (2) was employed to describe the isothermal crystallization kinetics:

(2) $$X(t)=1-\exp(-kt^{n})$$

where *n* is the Avrami exponent, which is a constant that depends on the nucleation mechanism and type of crystal growth, and *k* is the Avrami rate constant associated with nucleation and growth rate parameters. Equation (2) can be rewritten as:

(3) log[−ln(1−X(t))]=logk+nlogt

and the values of log [−ln(1 − *X*(*t*))] plotted versus log *t*, allowing the Avrami exponent *n* and the crystallization rate constant *k* to be calculated from the slope and intercept of the linear fit, respectively. It is rare that the Avarmi equation can be used to describe the entire crystallization process, but is widely accepted as valid at the early stage of crystallization, as previously reported in our studies for nylon-6/IF-WS_2_ [21] and PLLA/INT-WS_2_ [25]. Linear regressions of these straight lines at low levels of crystalline transformation (5–40%) yielded the Avrami exponents (*n*) shown in Table 1. As an example, Figure 2 illustrates the Avrami plots for both neat PHBV and PHBV/INT-WS_2_ (1.0 wt %) at different crystallization temperatures and the data are represented in Table 1. The average values obtained for *n* varied with the INT-WS_2_ concentration: *n* ≈ 3.0 for neat PHBV, *n* ≈ 3.3 for PHBV/INT-WS_2_ (0.1 wt %), *n* ≈ 4.2 for PHBV/INT-WS_2_ (0.5 wt %) and *n* ≈ 4.4 for PHBV/INT-WS_2_ (1.0 wt %). According to the ideal case of the Avrami equation for nucleated crystallization with three-dimensional crystal growth, the value of the exponent should be *n* = 3 [31]. A value of *n* = 4 indicates ideal three-dimensional growth with a linear increase in nucleation sites over time due to heterogeneous nucleation, which was expected for the binary composite materials with the addition of nanoparticles. However, the ideal state was not achieved during the crystallization process probably due to athermal crystallization and/or imperfections within the polymer network (entanglements, single chains transversing multiple lamellae, etc.) as well as secondary crystallization processes, mixed nucleation modes and the change in material density [3]. Moreover, even some experimental factors such as an error introduced in the determination of the onset of crystallization or induction time, the establishment of the baseline and incomplete isothermal crystallization data, the effect of the cooling rate from the melt to the isothermal crystallization temperature and the conversion range employed for the fitting can lead to non-integer values of *n* [8]. In addition, the supernucleation effect of the nanotubes could also be connected to the variation of the *n* exponents [9]. The values of *n* reported in the literature for PHBV systems are dispersed, ranging from 1.7 to 4 [3]. Liu et al. [32] also obtained *n* values of 2.0–2.2 for P(3HB-*co*-3HV) (6.6 mol % HV). Chan et al. [33], however, obtained *n* values across a range of HV contents of 2.35–2.7, and Saad et al. [34] obtained an exponent of 3.8 for thin P(3HB) films. Meanwhile, Xu et al. [35] applied the Avrami equation to IR data for isothermal crystallization of P(3HB) and Nodax (P(3HB-*co*-3HHx)) and obtained an *n* value of 1.72 and 2.08 respectively, indicating that a heterogeneous nucleation mechanism exists in this case. Nonetheless, the increase of the Avrami exponent compared to the neat sample did justify the fact that the initial nucleation stage was enhanced by the nanoparticles. With the linearized Avrami plots, the intercept value is ln(*k*) and the overall rate constant (*k*) is easily determined.

**Figure 3:**
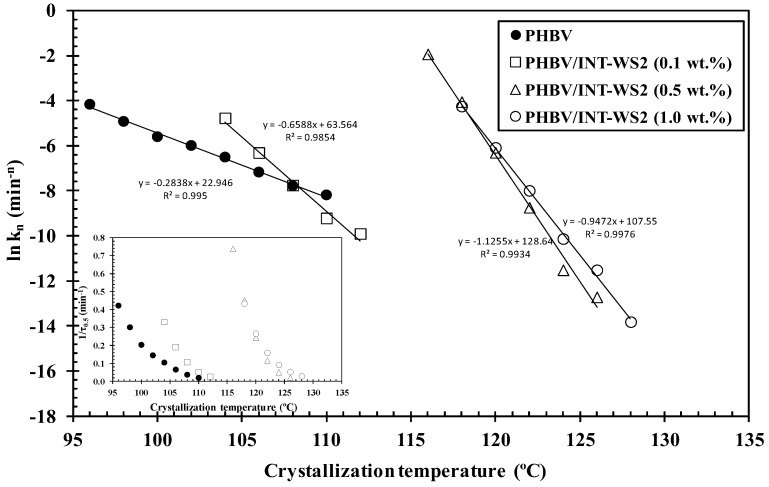
Logarithmic plots of the global rate constant (*k*) of PHBV/INT-WS_2_ nanocomposites as a function of the crystallization temperature (*T_c_*); the inset represents the inverse of crystallization half-time (1/*τ*_0.5_) as a function of *T_c_*.

**Figure 4:**
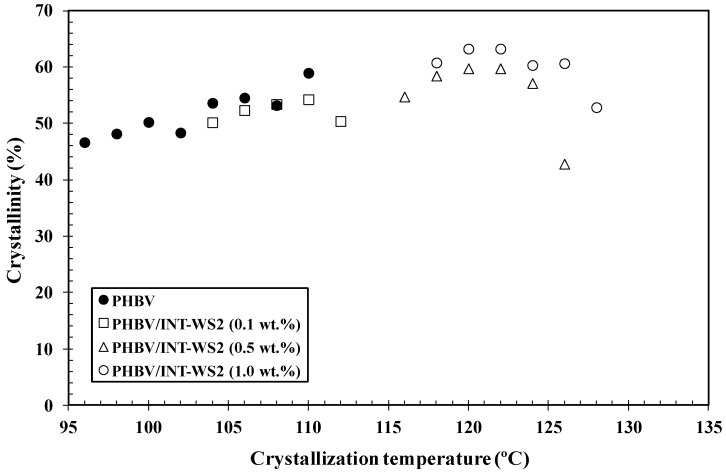
Variation of the crystallinity of isothermal crystallization of PHBV/INT-WS_2_ nanocomposites as a function of the crystallization temperature (*T_c_*).

Another common way to estimate the overall rate constant, also *k*, is by the well-fitting logarithmic representation of the following expression [36]:

(4) $$k_{n}=\frac{\ln2}{{\left(\tau_{0.5}\right)}^{n}}$$

where *τ*_0.5_ is the time needed to reach 50% crystalline transformation. Values of *k_n_* obtained for both neat PHBV and its nanocomposites are represented in Figure 3, where the effect of the crystallization temperature (*T_c_*) and INT-WS_2_ concentration on the overall crystallization rate can be observed (Table 1). By increasing *T_c_* from 96 °C to 110 °C, the crystallization of neat PHBV becomes hindered and *k_n_* decreases due to the excessive mobility of the polymer chains that reduces the development of nucleation sites at the higher temperature. A similar phenomenon takes place for PHBV/INT-WS_2_ (1.0 wt %) in the range of 118–128 °C. The values of *k_n_* for the nanocomposites were found to be higher in all cases than those for PHBV. Whilst PHBV presented a value of *k_n_* ≈ 2.51 × 10^−5^ at *T_c_* = 102 °C, in the case of the nanocomposites, the values of *k_n_* of around the same order were obtained at 120 °C for a concentration of 1.0 wt % of INT-WS_2_. The results show that INT-WS_2_ nanoparticles are effective nucleating agents for PHBV, promoting the nucleation of the crystallization of the polymer chains at small nanofiller loadings, without altering its crystal structure [29]. Additionally, the presence of increasing content of INT-WS_2_ also improves the crystallinity of PHBV, as can be seen in Figure 4.

**Figure 5:**
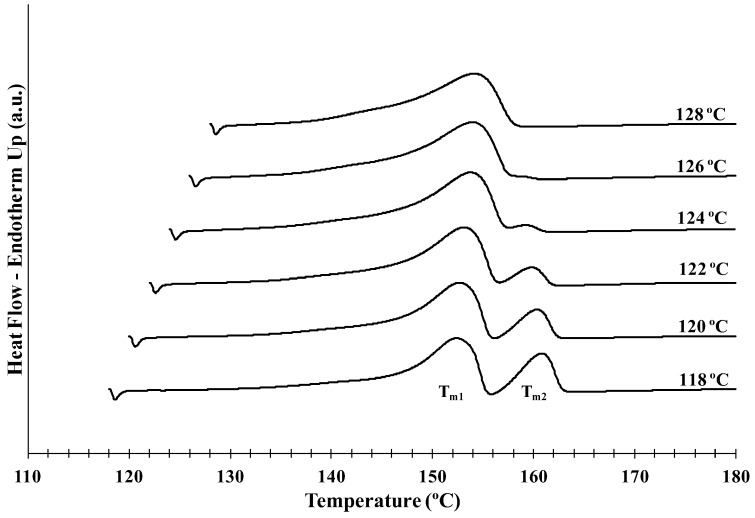
Melting DSC thermograms of PHBV/INT-WS_2_ (1.0 wt %) nanocomposite obtained at a heating rate of 5 °C min^−1^ after isothermal crystallization at the indicated temperatures.

Hereafter, the melting behavior of PHBV/INT-WS_2_ will be presented in order to understand the dependence of the double melting temperatures of PHBV with composition. Figure 5 shows the DSC melting curves obtained in this study after isothermal crystallization at different *T_c_* for PHBV/INT-WS_2_ (1.0 wt %), where a clear double melting behavior was observed and the results for all samples are summarized in Table 1. No clear difference was observed in the evolution of the double melting temperature with INT-WS_2_ due the large shift of the crystallization interval of PHBV to higher temperature and direct temperature comparisons were not feasible (i.e., the measurable temperature range for neat PHBV was 96–110 °C, whereas for the 1.0 wt % nanocomposite, the measurable temperature range was 118–128 °C).

### 3.2. Crystallization Activation Energy

**Figure 6:**
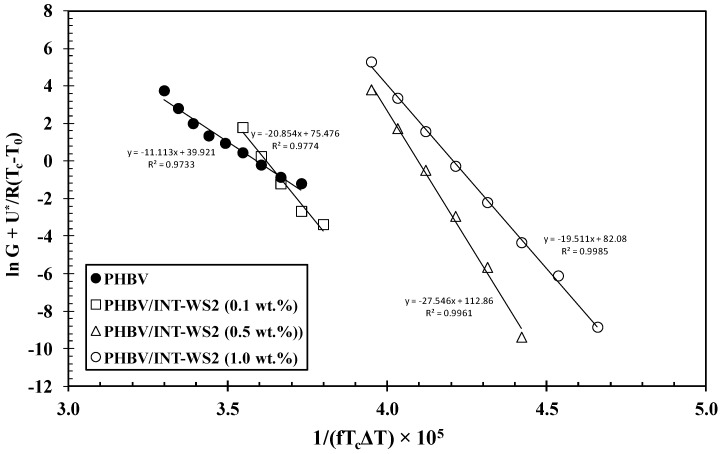
Logarithmic plots of Lauritzen and Hoffman (L–H) equation for PHBV/INT-WS_2_ nanocomposites.

For further insight into the crystallization behavior of PHBV and its nanocomposites, the free energy of folding (*σ_e_*) was calculated using the Lauritzen and Hoffman (L–H) model [37,38], previously adopted to calculate the isothermal crystallization activation energy of nylon-6/IF-WS_2_ [21] and PLLA/INT-WS_2_ [25]. In this approximation, *σ_e_* represents the energy required to fold the polymer chains during crystallization. In agreement with the kinetic theory of crystallization and independent regime type, the crystallization rate (*G*) can be expressed as:

(5) $$G=G_{0}\exp[-\frac{U^{*}}{R(T_{c}-T_{0})}]\exp[-\frac{K_{g(\mathrm{III})}}{fT_{c}\Delta T}]$$

where *G*_0_ is a temperature independent pre-exponential term, U* is the activation energy required for chain movement (2.45 × 10^6^ cal kmol^−1^), *T*_0_ is the temperature at which there is no chain motion (usually *T*_0_ = *T_g_* − 51.6 K), *R* is the universal gas constant, Δ*T* is the undercooling, or $T_{m}^{0}$ − *T_c_* where $T_{m}^{0}$ is the equilibrium melting temperature, *f* is a correction factor that accounts for the variation of the equilibrium melting enthalpy ($\Delta H_{m}^{0}$) with temperature, defined as 2*T_c_*/(*T_c_* + $T_{m}^{0}$), and *K_g_* is the nucleation constant for Regime III [3,39], which can be expressed by:

(6) $$K_{g(III)}=\frac{4b_{0}\sigma_{u}\sigma_{e}T_{m}^{0}}{k_{B}\Delta H_{m}^{0}}$$

where *k* is the Boltzmann constant, 1.38 × 10^−26^ kJ K^−1^, *b*_0_ = 7.2 nm and corresponds to the thickness of a single crystalline monolayer added during growth [3], and *σ_e_* and *σ_u_* are the basal and lateral interfacial free energies of the crystallite, respectively. The logarithmic representation of the first term of Equation (5) versus 1/*fT_c_*Δ*T* is presented in Figure 6 for all the samples analyzed, and the linear fits observed support unique regime behavior. The values of *K_g_*_(*III*)_ were calculated from the slopes of these plots.

Although the literature values found for Δ*H_m_* vary considerable since they are fundamentally conditioned by the determination method [3], the influence of this experimental variability in the comparative analysis of the values of the interfacial free energies can be eliminated by applying the Hoffman approximation [38], which determines the interfacial free energy using the following expression:

(7) $$\sigma_{u}=\alpha \Delta H_{m}^{0}\sqrt{a_{0}b_{0}}$$

where *α* = 0.24 (for high melting polyesters) and *a*_0_*b*_0_ = 38.01 Å [40] that corresponds to the chain cross-section in the PHBV crystal. Thus, the basal interfacial free energy can be derived from the following equation:

(8) $$\sigma_{e}=\frac{k_{B}K_{g(III)}}{4b_{0}T_{m}^{0}\alpha \sqrt{a_{0}b_{0}}}$$

Under these considerations and based on our previous studies of the isothermal crystallization of polymer/WS_2_ systems [21,25], the values of *σ_e_* obtained for PHBV, PHBV/INT-WS_2_ (0.1 wt %), PHBV/INT-WS_2_ (0.5 wt %) and PHBV/INT-WS_2_ (1.0 wt %) are around 75, 57, 71 and 58 erg cm^−2^, respectively. There is a clear trend for decreasing values of *σ_e_* as the INT-WS_2_ content is increased. From these results, we can conclude that between around 6–24% lower energy is required to generate crystalline nuclei of PHBV, which in turn also promotes the formation of new PHBV crystal surfaces. This excellent matching suggests that PHBV crystals might grow on the INT-WS_2_ surface by an epitaxial mechanism in absence of the chemical interaction between the INT-WS_2_ and PHBV. More in-depth experiments will be conducted to verify this proposal in a future study.

### 3.3. Spherulitic Growth Analysis from POM Observation

**Figure 7:**
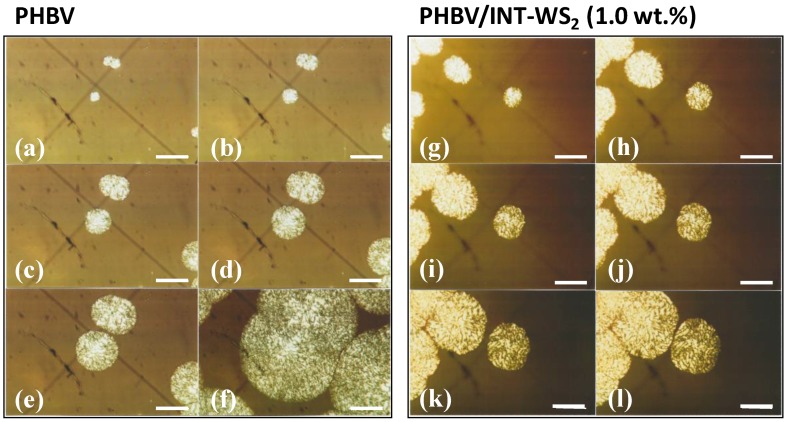
POM images of PHBV/INT-WS_2_ nanocomposites. PHBV: (**a**–**f**) are images taken at 8 min; 12 min; 23 min; 30 min; 37 min and after 60 min rapidly cooled to 80 °C, respectively; and PHBV/INT-WS_2_ (1.0 wt %): (**g**–**l**) are images taken at 4 min; 5 min; 6 min; 7 min, 8 min and 10 min, respectively. Scale bar 100 μm.

**Figure 8:**
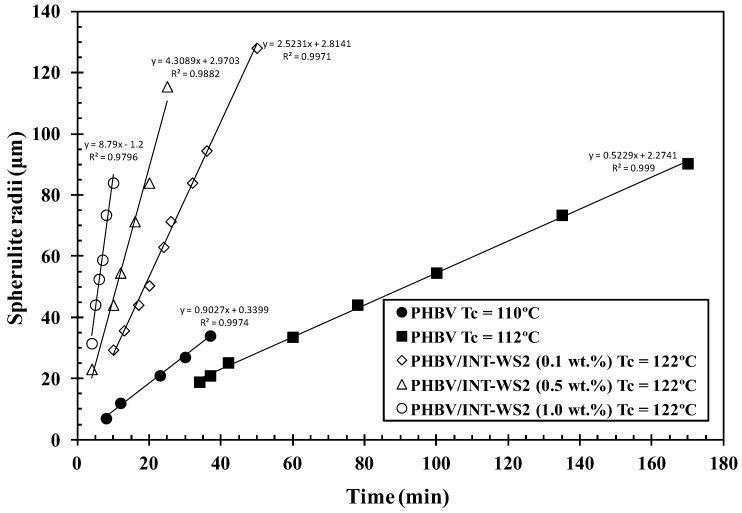
Isothermal spherulitic radii of PHBV/INT-WS_2_ nanocomposites obtained at the indicated crystallization temperatures.

Optical light microscopy images of crystals produced during the isothermal crystallization of PHBV and its nanocomposite containing INT-WS_2_ were obtained. As an example, Figure 7 and Figure 8 shows the spherulitic morphology of neat PHBV and PHBV/INT-WS_2_ (1.0 wt %) isothermally crystallized at 100 °C and 122 °C, respectively. 

The mainly circular superstructures are indicative of the formation of a central nucleus followed by radial, outward growth, as expected. In Figure 7e crystal diameters of approximately 130 μm were observed after 37 min and in Figure 7k crystal diameters of 140 μm were seen after only 8 min. This observation further confirms the acceleration of the crystallization process of PHBV by incorporation of INT-WS_2_. Another effect observed in the nucleated systems was an average decrease in crystal diameters of the neighboring spherulites before impingement. This was due to a higher density of nucleation sites and faster crystal formation compared to the neat copolymer. Assuming linear, constant growth rates, a linear slope of the growth diameter with respect to time for the four samples clearly indicated that crystal growth is faster in the samples containing INT-WS_2_ than in the pure PHBV sample (Figure 8). The crystal growth rates for neat PHBV are 0.54 and 0.92 μm min^−1^ at *T_c_* = 110 °C and 112 °C, respectively. The crystal growth rates for the 0.1, 0.5 and 1.0 wt % nanocomposites are 2.61, 4.48, and 8.62 μm min^−1^, which is sixteen times faster for the 1.0 wt % sample compared to the neat polymer at the two different *T_c_* values. Another way to interpret this figure is by comparing the time required for the spherulites to reach a certain diameter. To reach a diameter of, for example, 35 μm at *T_c_* = 122 °C, inorganic nanotube fractions of 1.0 wt %, a 0.5 wt %, and 0.1 wt % in PHBV, needed approximately 4, 8, and 13 min, respectively. However, a neat PHBV sample requires around 38 and 65 min for *T_c_* = 110 °C and 112 °C, respectively. This is another clear indication of the increase in the crystallization rate of PHBV due to the incorporation of the well-dispersed INT-WS_2_ nanofiller. In particular, the increasing slope of the growth rate of the spherulite radii as a function of INT-WS_2_ concentration may be due to the increased nucleation sites available thus facilitating accelerated growth in either case. More research would be required. 

## 4. Conclusions

From the melt-crystallization measurements, it was shown that INT-WS_2_ strongly affect the crystallization of PHBV polymer. INT-WS_2_ accelerate the crystallization process of PHBV in the nanocomposites and shift the crystallization temperature/interval for PHBV to higher temperatures with increasing INT-WS_2_ content. The experimental data fitted very well to the Avrami kinetic model. In particular, it was found that the value of the Avrami exponent *n* for PHBV/INT-WS_2_ nanocomposites increased compared to that for neat PHBV, and the analysis of the activation energy of crystallization regime III using the Lauritzen and Hoffman (L–H) model showed that for the PHBV/INT-WS_2_ nanocomposites, the fold surface free energy (*σ_e_*) of PHBV chains decreased with increasing INT-WS_2_ content. Similarly, the variation of the spherulitic radii of PHBV with crystallization time and concentration of nanofiller, calculated from the POM micrographs, also supports the spherulite growth-accelerating effect of PHBV. While the crystallization occurred at much higher temperatures with the incorporation of INT-WS_2_, the direct comparisons of crystallization rates were not quantifiable at equivalent temperatures because of the acceleration effect of the doped materials. This new knowledge obtained from the crystallization kinetics of the PHBV biopolymer and its nanocomposites can provide an essential benchmark for optimizing the design and processing of PHBV-based thermoplastic materials with desirable properties. 

## Figures and Tables

**Table 1:** Isothermal crystallization parameters of PHBV and PHBV/INT-WS_2_ nanocomposites. *T_c_* = crystallization temperature, *τ*_0.5_ = the time needed to reach 50% crystalline transformation, *n* = Avrami exponent, *k_n_* = overall rate constant, *T_m_* = melting temperature and *σ_e_* = fold surface free energy. (*) The main error arises from baseline selection during data processing. We estimate the following errors: ±1 J/g for (Δ*Ht*) and ±100 for Δ(*τ*_0.5_).

INT-WS_2_ Content (wt %)	*T_c_* (°C)	*τ*_0.5_ (min)	*n*	*k_n_* × 10^5^	*T_m_*_1_/*T_m_*_2_ (°C)	*σ_e_* (erg cm^−2^)
0	96	2.4	4.4 ± 0.1	15.65	149.6/160.0	75 ± 3
	98	3.3	3.8 ± 0.1	7.31	148.5/159.5	
	100	4.9	3.3 ± 0.1	3.71	147.5/158.0	
	102	6.9	2.9 ± 0.2	2.51	147.1/157.3	
	104	9.5	2.7 ± 0.1	1.5	146.9/156.8	
	106	15.1	2.5 ± 0.1	0.77	145.7/154.9	
	108	26.3	2.3 ± 0.1	0.42	144.7/153.6	
	110	49.3	2.0 ± 0.2	0.28	145.2/155.8	
0.1	104	3	4.0 ± 0.1	8.49	145.2/155.8	57 ± 2
	106	5.2	3.6 ± 0.1	1.83	144.2/154.4	
	108	9.3	3.3 ± 0.2	0.43	144.1/153.7	
	110	19	3.0 ± 0.1	0.1	144.4/153.0	
	112	37.2	2.7 ± 0.1	0.05	144.8/152.3	
0.5	116	1.4	5.2 ± 0.1	145.23	156.7/165.2	71 ± 3
	118	2.2	4.6 ± 0.2	17.5	154.6/165.2	
	120	4.1	4.2 ± 0.1	1.84	153.6/161.2	
	122	8.6	3.9 ± 0.1	0.16	153.3/160.0	
	124	19.5	3.7 ± 0.2	0.01	153	
	126	47.6	3.8 ± 0.2	0.003	152.5	
1	118	2.3	4.6 ± 0.1	14.37	152.4/160.8	58 ± 2
	120	3.8	4.3 ± 0.1	2.29	152.7/160.3	
	122	6.3	4.1 ± 0.1	0.34	153.1/160.0	
	124	10.8	4.1 ± 0.2	0.04	153.8/160.3	
	126	19	3.9 ± 0.2	0.01	154	
	128	34.9	4.0 ± 0.2	0.005	154.7	
